# Mitochondrial membrane potential played crucial roles in the accumulation of berberine in HepG2 cells

**DOI:** 10.1042/BSR20190477

**Published:** 2019-04-26

**Authors:** Qiao Li, Ting Zhou, Chang Liu, Xiao-Yu Wang, Ji-Quan Zhang, Fei Wu, Ge Lin, Yue-Ming Ma, Bing-Liang Ma

**Affiliations:** 1 Department of Pharmacology, School of Pharmacy, Shanghai University of Traditional Chinese Medicine, Shanghai, China; 2 Department of Gastroenterology, Shuguang Hospital Affiliated to Shanghai University of Traditional Chinese Medicine, Shanghai, China; 3 Experiment Center for Science and Technology, Shanghai University of Traditional Chinese Medicine, Shanghai, China; 4 Engineering Research Centre of Modern Preparation Technology of TCM of Ministry of Education, Shanghai University of Traditional Chinese Medicine, Shanghai, China; 5 School of Biomedical Sciences, The Chinese University of Hong Kong, Hong Kong SAR, China

**Keywords:** berberine, cellular pharmacokinetics, HepG2, mitochondria membrane potential, subcellular distribution

## Abstract

Berberine is a natural alkaloid that has antineoplastic effects. However, in hepatoma cells like HepG2, the expressions of uptake transporters are minimal but efflux transporters are relatively high. Hence, how berberine enters and reaches a cytocidal concentration remains to be elucidated. In the present study, we revealed the accumulation mechanism of berberine in HepG2 cells. Cell organelles were isolated based on differential centrifugation; berberine concentration was measured using a liquid chromatography-tandem mass chromatography method or flow cytometry. Subcellular distribution of berberine was observed using a laser scanning confocal microscopy. The results showed that berberine was concentration-, temperature-, and time-dependently taken up and accumulated in HepG2 cells. Membrane drug transporters and cell membrane potential had limited effects in berberine uptake. However, qualitative and quantitative studies showed that berberine was enriched in the mitochondria; inhibition of mitochondrial membrane potential (MMP) by carbonyl cyanide 3-chlorophenylhydrazone (CCCP) significantly decreased the intracellular berberine by up to 70%. More importantly, MMP not only significantly enhanced berberine uptake driven by cell membrane potential (*P*<0.01) but also inhibited p-glycoprotein (P-gp)-mediated berberine efflux (*P*<0.01). In brief, our results for the first time showed that MMP played crucial roles in berberine accumulation in HepG2 cells.

## Introduction

Berberine is a natural quaternary protoberberine alkaloid [1]. It has various pharmacological effects including antimicrobial, antioxidant, anti-inflammatory, anti-cholesterol, and antidiabetic activities [2]. Clinical trials indicate that berberine has potential therapeutic effects on type 2 diabetes mellitus and hyperlipidaemia with no serious side effects [3].

Given its extremely low blood concentration after administration, it is once confusing how oral berberine could exert such wide and significant *in vivo* pharmacological effects. The maximum plasma concentration (C_max_) of berberine varies from several to more than a dozen nanograms per millilitre in experimental animals and 0.4 ng/ml in volunteers [4]. The bioavailability of oral berberine is as low as 0.36% [5], mainly because of its limited solubility [6], extensive first-pass metabolism [5], and efflux mediated by p-glycoprotein (P-gp) during intestinal absorption [7] and after hepatic distribution [8]. However, relatively high tissue distribution, especially hepatic distribution of berberine provides evidence for its lipid and glucose lowering activities [5]. Human organic cation transporter (OCT) 1 (OCT1) and organic anion-transporting polypeptides (OATP1B3) contribute to berberine hepatic distribution [9,10]. The addition of silymarin, which inhibits P-gp, is able to improve the therapeutic effects of oral berberine on lipid and glucose metabolism by improving its bioavailability [11].

Berberine also shows antineoplastic effects [12]. It induces G_1_-phase cell cycle arrest [13], caspase-3/mitochondria-dependent apoptosis [14], and autophagic cell death [14] in cancer cells. However, in terms of cancer cells such as HepG2 cells that are widely used to study the anti-hepatoma effects of berberine [15], the expression of uptake transporters [for example, OCT1 and OATP1B3 (i.e. OATP8)] is minimal but efflux transporter (for example, P-gp) is very high [16]. Hence, how berberine enters and reaches a concentration that is high enough to kill the hepatoma cells remains to be elucidated.

As an alkaloid [1], berberine may be taken up by mitochondria and/or lysosomes. In terms of mitochondria, scientists have known the involvement of mitochondrial membrane potential (MMP) in the cellular uptake and retention of lipophilic cationic agents [17]. Given that there will be a ∼10-fold distribution of cations within mitochondria for every ∼60 mV increase in MMP and the MMP is typically 140–180 mV, there will be a several hundred-fold distribution of cations into the mitochondrial matrix [18]. Actually, berberine could be taken up by mitochondria [19,20]. The reports are of great significance in describing the subcellular distribution of berberine, but the roles of such distribution in the cellular accumulation of berberine are still unclear. In addition, studies reveal that lipophilic or amphiphilic compounds with a basic moiety can enter lysosomes and get protonated owing to their weak base properties, and can be reserved within lysosomes owing to the mechanism of ion trapping [21]. Lysosomal distribution may lead to the cellular transfer of drugs against a huge concentration gradient [22]. Taking the lysosomotropic drug quinacrine as an example, its lysosomal concentration is approximately 750 times greater than its extracellular concentrations [23]. However, lysosomal distribution of berberine has not been reported, let alone its roles in cellular berberine accumulation.

Therefore, we studied the cellular accumulation mechanisms of berberine in HepG2 cells, which would contribute to reveal its potential site and mechanism of action.

## Materials and methods

### Materials

Berberine chloride, carbamazepine, and verapamil with purities of more than 98% were purchased from the National Institute for the Control of Pharmaceutical and Biological Products (Beijing, China). Rifampicin and cimetidine, with purities of more than 98%, were obtained from the Shanghai Yuanye Biotechnology (Shanghai, China). Mitochondria isolation kits for cultured cells, carbonyl cyanide 3-chlorophenylhydrazone (CCCP, purity ≥97%), rhodamine 123 (Rho123), culture-grade dimethyl sulphoxide (DMSO), HEPES-free acid, and acetonitrile, were the products of the Merck Life Science (Shanghai) (Shanghai, China). Foetal bovine serum (FBS), the modified Eagle’s minimum essential medium (MEM), Mitotracker™ deep red FM-special packaging, and Lysotracker™ deep red were the products of Thermo Fisher Scientific (MA, U.S.A.). Trypsinase and penicillin–streptomycin solution were obtained from Biosharp (Hefei, China). The Quantipro Bicinchoninic Acid (BCA) assay kit was obtained from the Beyotime Biotechnology (Shanghai, China). Pure water used in the current study was prepared using a Milli-Q system (Millipore Corporation, Billerica, MA, U.S.A.). All other materials were of analytical grade or better.

### Cell culture

Human hepatoblastoma cell line HepG2 was purchased from and authenticated by the Chinese Academy of Sciences Cell Bank (Shanghai, China). The cells were cultured at 37°C in MEM supplemented with 10% FBS, penicillin–streptomycin solution, and HEPES (15 mM) in a humidified atmosphere of 5% CO_2_ and subcultured when approximately 85% confluence was achieved with trypsinase solution (0.25%, dissolved in PBS, i.e. phosphate buffer). The cells with passage numbers between 3 and 20 were used.

To avoid the potential influences of FBS and other components in the MEM culture medium on the subcellular transfer of berberine, HBSS [consisting of (in mM) 135 NaCl, 1.2 MgCl_2_, 0.81 MgSO_4_, 27.8 glucose, 2.5 CaCl_2_, and 25 HEPES, pH 7.2] [24], but not the regular culture medium (i.e. the FBS containing MEM culture medium), was used in the following drug incubation experiments.

Berberine was dissolved in DMSO and diluted with HBSS in the study. To avoid the potential influences of DMSO on cell viability, the concentration of DMSO in the culture medium was lower than 1% and in most situations was restricted to 1%.

### Liquid chromatography tandem mass spectrometry

Briefly, a Shimadzu Prominence UFLCXR series HPLC (Shimadzu, Japan) and a Thermo Scientific TSQ Quantum Ultra mass spectrometer (Thermo Scientific, Waltham, MA, U.S.A.) equipped with an electrospray ionisation (ESI) source were used. Carbamazepine was used as an internal standard. The samples were precipitated with three volumes of acetonitrile. After centrifugation (25000×***g***, 10 min, 4°C), the supernatant was mixed with an equal volume of water, and 10-μl samples were injected into the liquid chromatography tandem mass spectrometry (LC-MS/MS) system. The samples were eluted through a Hypersil Gold (C18) analytical column (5 µm, 100 × 2.1 mm) with a gradient of the aqueous phase (0.08% v/v formic acid and 2 mM ammonium acetate) and the acetonitrile phase (0 min, 85:15; 7 min, 32:68; 7.01 min, 85:15; 10 min, 85:15) at a flow rate of 0.3 ml/min. The ESI source was set to positive ion mode. Data acquisition was performed in the multiple reaction-monitoring mode of the selective mass transition for each compound. The transitions from the precursor ions to the protonated fragment product ions were monitored as follows: *m/z* 336.2 to *m/z* 322.3 for berberine, and m/z 237.00 to m/z 194.31 for carbamazepine. The linear dynamic range for berberine was 1.95–1000 ng/ml, which met the requirements of quantitative determination of berberine in the tested biological samples. The quality control samples were prepared at three different concentrations. The accuracy, precision, recovery, and stability tests all met the requirements of quantitative determination in biological samples, and the method was used in our previous studies [6,25].

### Flow cytometry analysis

The concentration of berberine and Rho123 (a organic cation used to reflex the MMP in the study) in the cells were semi-quantified by flow cytometry analysis (Becton-Dickenson FACSCalibur™ system, New Jersey, U.S.A.) according to a previous report [18]. For berberine or Rho123, the excitation wavelength is at 488 or 507 nm and the emission wavelength is at 530 or 529 nm, respectively. Briefly, after incubation with berberine or Rho123, the drug-containing HBSS was removed and the cells were washed with chilled PBS for three cycles and harvested by trypsinisation. After centrifugation (1000 rpm, 4°C, 2.5 min), the pellet was resuspended in 1 ml chilled PBS and immediately measured using the flow cytometer. In each experiment, the obtained fluorescence intensity of the treated cells was normalised by deducting the fluorescence intensity of the blank cells. In some cases, the obtained fluorescence intensity of the treated cells was normalised by comparing with the fluorescence intensity of the control cells. For all samples, the time lag between the removal of the drug-containing HBSS and the measurement of fluorescence intensity was less than 20 min. The fluorescence intensity was verified to be stable during this period.

### Accumulation of berberine in the HepG2 cells

The cells were incubated with berberine (1 or 10 μM) at 37°C for different times [0.033 (only for 10 μM), 0.125, 0.25, 0.5, 1, 2, 4, or 6 h]. The incubation medium was collected at the designated time points. The cells were then washed three times with chilled PBS and harvested by trypsinisation. The cells were stored in 0.5 ml chilled water and got broken by repeated freezing and thawing. The concentration of berberine in the incubation medium and the cell homogenate were quantified using the LC-MS/MS method. The ratios of the intracellular to extracellular (i.e., the cell homogenate to incubation medium) concentration of berberine were calculated to reflex the cellular accumulation of berberine.

### Uptake of berberine by the HepG2 cells

For the time-dependent uptake experiment, HepG2 cells were incubated with 30 μM berberine at 37°C for different times, respectively. For the dose- and temperature-dependent uptake experiments, HepG2 cells were incubated with 0.9, 3, 9, 30, 90, or 300 μM berberine at 4 or 37°C for 0.5 h, respectively. The incubation of berberine at 30 μM for 6 h or at concentration up to 300 μM for 0.5 h did not cause cytotoxicity in the cells according to MTT assays (data not shown). To study the effects of transporter inhibitors on the uptake of berberine by the HepG2 cells, the cells were incubated with 10 μM berberine (as control), or in the presence of cimetidine [0.3, 1 mM, a typical OCTs inhibitor] or rifampicin [10 μM, a typical organic anion polypeptide transporters (OATPs) inhibitor] at 37°C for 0.5 h. To study the effects of cellular membrane potential on the uptake of berberine by the HepG2 cells, the cells were incubated with the HBSS or sodium-free HBSS (HBSS-K, sodium in HBSS was replaced with potassium [24]) containing 10 μM berberine at 37°C for 0.5 h. To study the synergistic effects between drug transporters, cellular membrane potential, and MMP, the cells were incubated with 10 μM berberine, with or without 0.125 μM CCCP, 0.3 or 1 mM cimetidine, or the HBSS-K solution at 37°C for 0.5 h. To study the effect of CCCP pretreatment on cellular concentration of berberine or Rho123 (an MMP indicator), HepG2 cells were pre-incubated with drug-free HBSS or HBSS containing 0.125, 0.25, 0.5, 1, or 2 μM CCCP at 37°C for 20 min; then equal volume of HBSS containing 20 μM berberine or 16 μM Rho123 was added and the cells were futher incubated at 37°C for 0.5 h. After incubation, the cells were washed three times with chilled PBS and harvested by trypsinisation. The cells were resuspended in 0.5 ml chilled PBS and the concentration of berberine in the cells was quantified by the flow cytometry method.

### Laser scanning confocal microscope observation of the subcellular distribution of berberine in the HepG2 cells

The subcellular distribution of berberine in the HepG2 cells was qualitatively observed using a laser scanning confocal microscope (LSCM) system (LEICA SP8, Wetzlar, Germany), according to a previously described protocol [19]. Briefly, the HepG2 cells seeded into 35-mm glass-bottom Petri dishes (Wuxi NEST Biotechnology, Wuxi, China) were stained with 1 ml mito-tracker red (25 nM) for 30 min or lyso-tracker red (200 nM) for 15 min, which are able to visualise mitochondria and lysosomes, respectively. The dye was then removed and the cells were washed three times with warmed HBSS (37°C). The cells were incubated with 30 μM berberine for 15, 45, or 60 min, respectively. The fluorescence of berberine (green), mitochondria (red), or lysosome (red) in the cells was observed using the LSCM system. Laser excitation and PMT gain were set at values to best visualise the range of fluorescence emitted by the cultures. For berberine, mito-tracker red, or lyso-tracker red, the excitation wavelength is at 488, 640, or 647 nm, and the emission wavelength is at 530, 662, or 668 nm, respectively.

### LC-MS/MS detection of berberine in the mitochondria of the HepG2 cells

The HepG2 cells were incubated with 10 μM berberine at 37°C for 0.25, 0.5, 1, 2, 4, or 6 h, respectively. After incubation, the cells were washed three times with chilled PBS and harvested by trypsinisation. The cells were stored in 0.5 ml chilled water and were broken by repeated freezing and thawing. The concentrations of berberine in the whole cells or the mitochondria isolated according to the manufacturer’s instructions were quantified using the LC-MS/MS method. The purity and integrity of the isolated mitochondria were observed using the LCSM method after the mitochondria were incubated with Rho123 solution (8 μM) for 30 min (data not shown). The concentration of berberine was calibrated according to the protein content of corresponding cell or mitochondria samples.

### Efflux of berberine out of HepG2 cells

The HepG2 cells were pre-incubated with 10 μM berberine at 37°C for 2 h; then the cells were incubated with drug-free HBSS or HBSS containing CCCP (0.25 μM), verapamil (100 μM, a typical P-gp inhibitor), or CCCP (0.25 μM) plus verapamil (100 μM) at 37°C for 0.5, 1, 2, 4 h, respectively. After incubation, the cells were washed three times with chilled PBS and harvested by trypsinisation. The cells were stored in 0.5 ml chilled PBS and the accumulated berberine in the cells was quantified by the flow cytometry method. The amount of berberine was normalised to that at zero time point.

### Statistical analysis

In the dose-dependent uptake experiments, the kinetic parameters were calculated with reference to the literature [26] and with the aid of the GraphPad Prism 5.0 software (GraphPad Prism Software, San Diego, CA). Briefly, the data were fitted to a modified Michaelis–Menten model, which was combined with a non-saturable process: V = *V*_max_ × [S]/(*K*_m_ + [S]) + P_dif_ × [S], where V is the uptake velocity of the substrate; [S] is the substrate concentration in the medium; *K*_m_ is the Michaelis–Menten constant; *V*_max_ is the maximum uptake rate of the saturable uptake; and P_dif_ is the non-saturable uptake clearance.

Data were expressed as mean ± S.D. Each experiment was repeated at least twice with a minimum of three replicates each time. Student’s *t* test was used to compare a single treatment mean with a control mean. Statistical significance was determined with one-way or two-way analysis of variance (ANOVA) for multiple comparisons. *P*<0.05 was considered significant, *P*<0.01 was considered highly significant.

## Results

### Accumulation of berberine in the HepG2 cells

**Figure 1:**
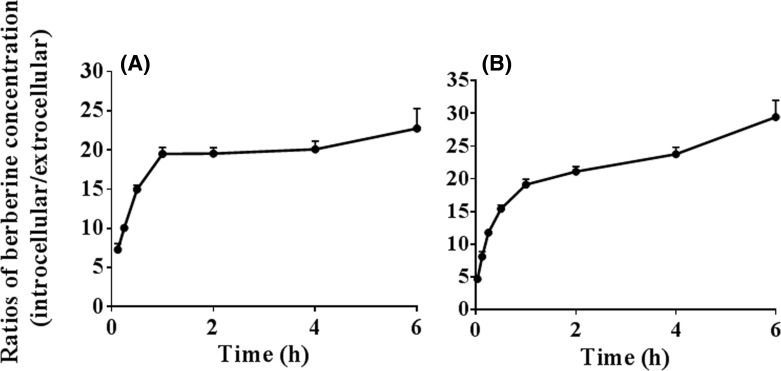
Accumulation of berberine in the HepG2 cells (Mean ± S.D., *n*=3) The cells were incubated with berbrine [1 (**A**) or 10 μM (**B**)] at 37°C for different times [0.033 (only for 10 μM), 0.125, 0.25, 0.5, 1, 2, 4, or 6 h]. The incubation medium was collected at the designated time points. The cells were then washed three times with chilled PBS and harvested by trypsinisation. The cells were stored in 0.5 ml chilled water and were broken by repeated freezing and thawing. The concentration of berberine in the incubation medium and the cell homogenate was quantified using the LC-MS/MS method. The ratios of the intracellular to extracellular (i.e., the cell homogenate to incubation medium) concentration of berberine were calculated to reflex the cellular accumulation of berberine.

The results in Figure 1 showed that the ratios of the intracellular to extracellular concentration (i.e., the cell homogenate to incubation medium concentration) of berberine increased with increased incubation time. After incubation for 0.5 h, the ratios reached above 15 in both low (1 μM) and high (10 μM) concentrations of berberine-treated groups. The results indicated that berberine was accumulated in the HepG2 cells. In addition, the ratios were independent of berberine concentrations in the incubation medium.

### Uptake of berberine by the HepG2 cells

**Figure 2:**
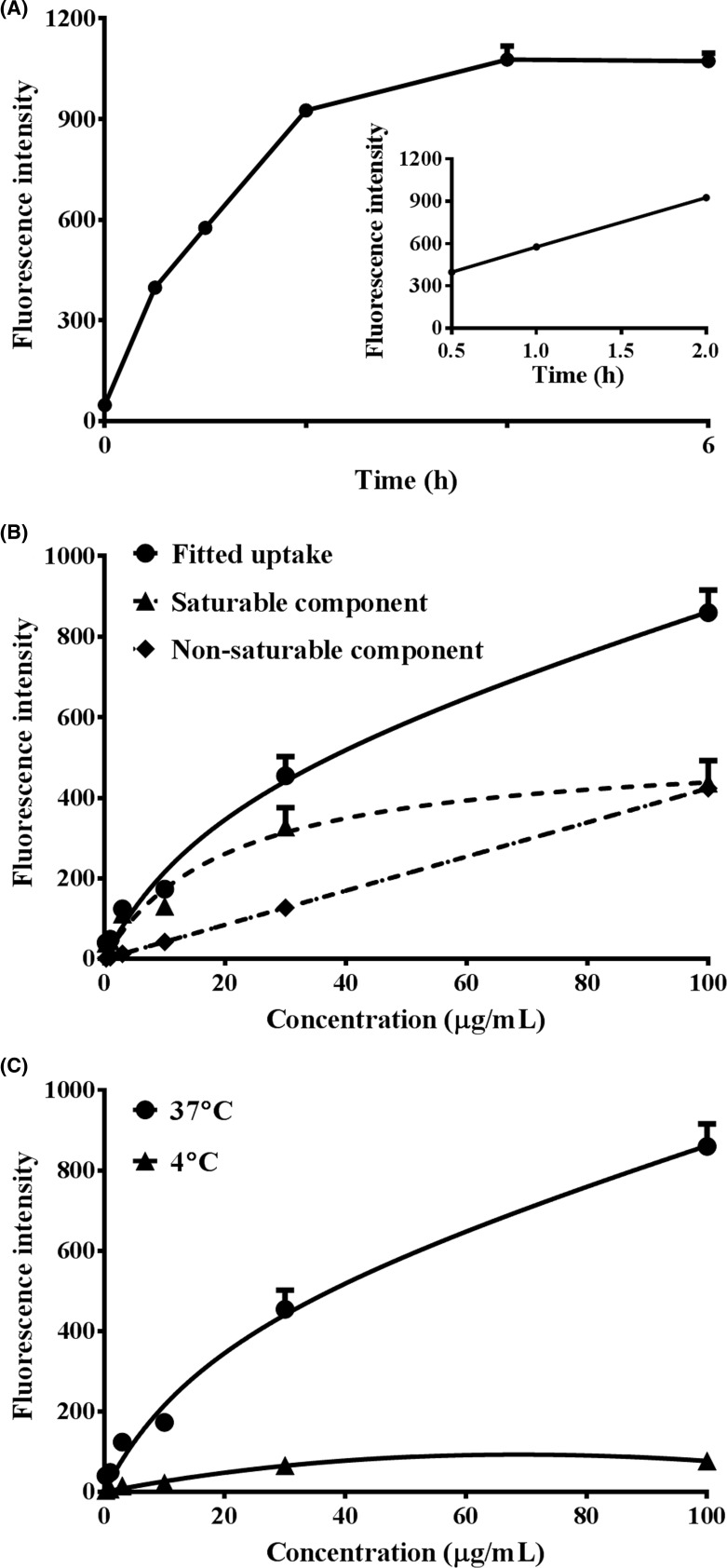
Time, dose, and temperature dependent uptake of berberine into HepG2 cells (Mean ± S.D., *n*=3) (**A**) HepG2 cells were incubated with 30 μM berberine at 37°C for 0.5, 1, 2, 4, or 6 h, respectively. (**B**) HepG2 cells were incubated with 0.9, 3, 9, 30, 90, or 300 μM berberine at 37°C for 0.5 h, respectively. (**C**) HepG2 cells were incubated with 0.9, 3, 9, 30, 90, or 300 μM berberine at 4 or 37°C for 0.5 h, respectively. After incubation, the cells were washed three times with chilled PBS and harvested by trypsinisation. The cells were stored in 0.5 ml chilled PBS and the accumulated berberine in the cells was semi-quantified by flow cytometry analysis with excitation at 488 nm and emission at 530 nm.

Berberine was taken up by the HepG2 cells in a time- (A), concentration- (B), and temperature-dependent (C) manner (Figure 2). To ensure the linearity of the uptake of berberine, the incubation time was hence designated at 0.5 h, and the concentration of berberine was set below 61.2 μM (the apparent *K*_m_ value calculated following the concentration-dependent uptake experiment) in subsequent experiments.

**Figure 3:**
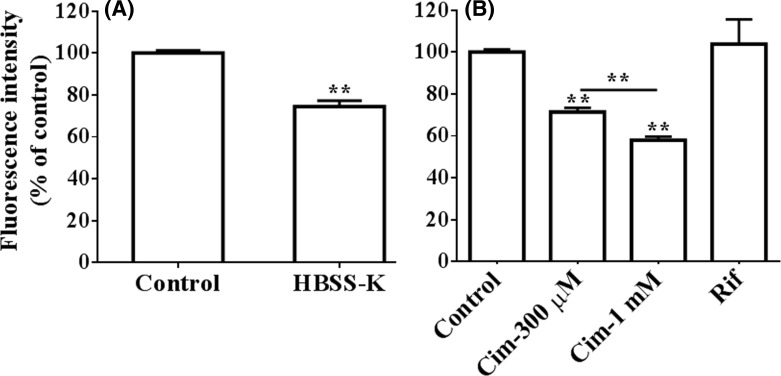
Influences of transporter and cell membrane potential on the uptake of berberine into HepG2 cells (Mean ± S.D., *n*=3) (**A**) HepG2 cells were incubated with HBSS or HBSS-K containing 10 μM berberine at 37°C for 0.5 h, respectively. (**B**) HepG2 cells were incubated with 10 μM berberine or in the presence of cimetidine (0.3, 1 mM, Cim) or rifampicin (10 μM, Rif) at 37°C for 0.5 h, respectively. After incubation, the cells were washed three times with chilled PBS and harvested by trypsinisation. The cells were stored in 0.5 ml chilled PBS and the accumulated berberine in the cells was quantified by flow cytometry analysis with excitation at 488 nm and emission at 530 nm. ** indicates *P*<0.01 *versus* control or between 0.3 or 1 mM cimetidine-treated groups.

As shown in Figure 3A, after decreasing the transmembrane potential difference by incubating the cells with the modified HBSS (sodium in HBSS was replaced with potassium), the intracellular berberine concentration decreased by 25.6 ± 2.8 % (*P*<0.01).

As shown in Figure 3B, 300 μM or 1 mM cimetidine decreased berberine concentration in the cells by 28.6 ± 2.0 or 42.1 ± 1.7% (both *P*<0.01), respectively. The results indicated that the role of OCTs in berberine accumulation was significant but limited. The results showed that rifampicin had no significant effect on berberine concentration (*P*>0.05) and hence excluded the contribution of OATPs in the uptake of berberine.

### Subcellular distribution of berberine in the HepG2 cells

**Figure 4:**
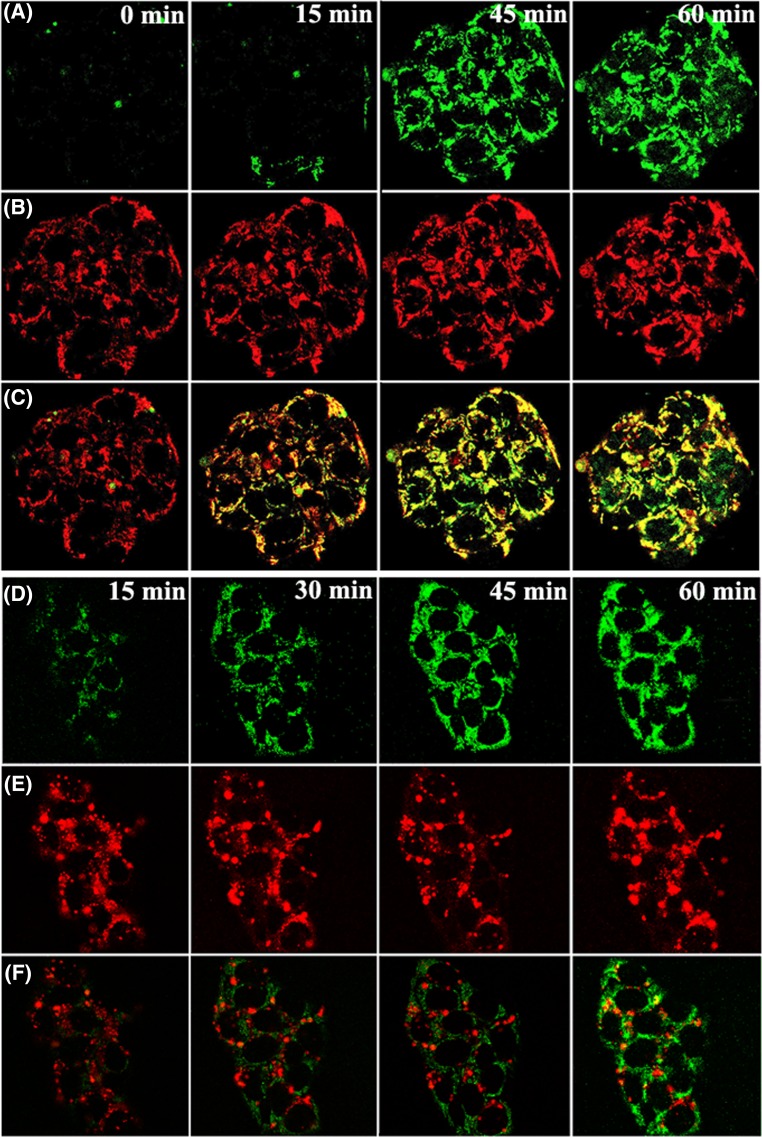
LSCM observation of the subcellular distribution of berberine in HepG2 cells HepG2 cells were stained with 1 ml mito-tracker red (25 nM) for 30 min or lyso-tracker red (200 nM) for 15 min, which are able to visualise mitochondria and lysosomes, respectively. The dye was removed and the cells were washed three times with warmed HBSS (37°C). Then the cells were incubated with 30 μM berberine for 15, 45, or 60 min. The fluorescence of beberine (green, **A**,**D**), mitochondrial (red, (**B**)), and lysosome (red, (**E**)) in the cells were observed using LSCM. For berberine, mito-tracker red, or Lyso-tracker red, the excitation wavelength is at 488, 640, or 647 nm and the emission wavelength is at 530, 662, or 668 nm, respectively. The stack of the green and red fluoresce marking mitochondria (**C**) or lysosome (**F**) was performed.

The green florescence (A and D) in Figure 4 increased with the incubation duration increasing from 0 to 60 min, which indicated the entry of berberine; red fluoresce, which was used to label mitochondria (B) or lysosome (E), was stable during this period. The stack of the green and red fluoresce marking mitochondria led to crescendo yellow fluoresce (C), which indicated the accumulation of berberine in mitochondria. However, the poor stack of the green and red fluoresce that marked lysosome (F) indicated the absence of berberine in lysosomes.

**Figure 5:**
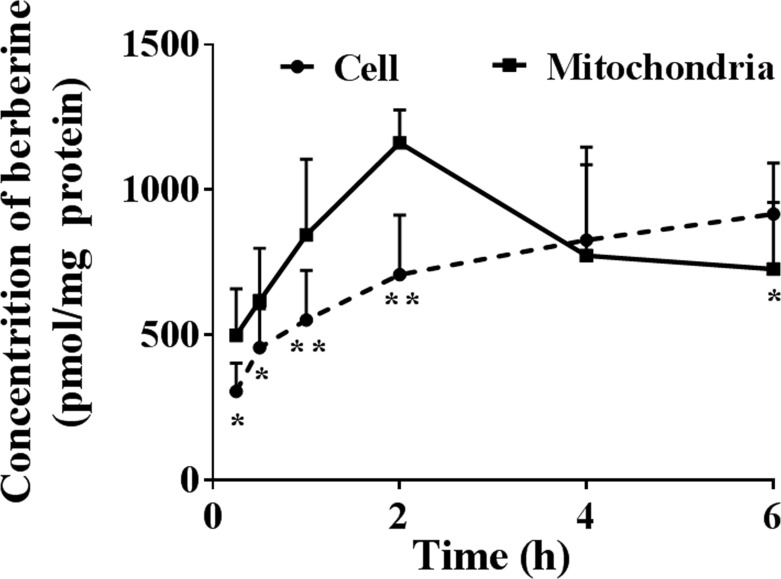
LC-MS/MS detection of berberine in the mitochondria of HepG2 cells (Mean ± S.D., *n*=3) HepG2 cells were incubated with 10 μM berberine at 37°C for 0.25, 0.5, 1, 2, 4, or 6 h, respectively. After incubation, the cells were washed three times with chilled PBS and harvested by trypsinisation. The cells were stored in 0.5 ml chilled water and were broken by repeated freezing and thawing. The concentration of berberine in whole cells or mitochondria isolated according to the manufacturer’s instructions was quantified using the LC-MS/MS method. The concentration of berberine was calibrated according to protein content. * indicates *P*<0*.*05 while ** indicates *P*<0.01 *versus* mitochondria.

The isolated mitochondria had satisfactory purity and integrity after isolation. The concentration of berberine in the isolated mitochondria was significantly higher than the average concentration of berberine in whole cells after 2 h of incubation (Figure 5), which verified the selective mitochondrial distribution of berberine. However, when the cells were incubated for a longer duration, mitochondrial berberine decreased, which indicated its redistribution from mitochondria.

### Influence of MMP inhibition on berberine accumulation in the HepG2 cells

**Figure 6:**
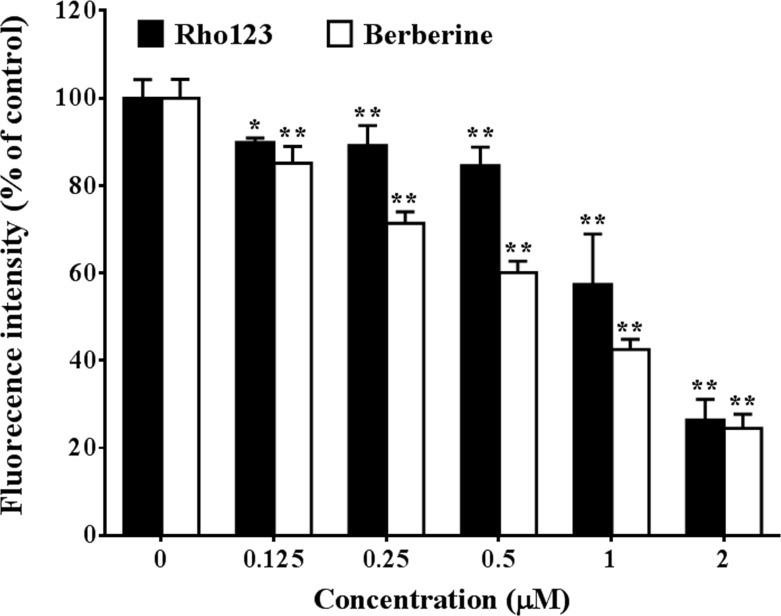
Influence of MMP inhibition on berberine accumulation in HepG2 cells (Mean ± S.D., *n*=3) HepG2 cells were pre-incubated with drug-free HBSS or HBSS containing 0.125, 0.25, 0.5, 1, or 2 μM CCCP at 37°C for 20 min, then equal volume of HBSS containing 20 μM berberine or 6 μg/ml Rho123 was added and the cells were further incubated at 37°C for 0.5 h. After incubation, the cells were washed three times with chilled PBS and harvested by trypsinisation. The cells were stored in chilled PBS and the concentration of berberine in the cells was quantified by flow cytometry analysis with excitation at 488 nm and emission at 530 nm. * indicates *P*<0*.*05 while ** indicates *P*<0.01 *versus* groups that treated without CCCP.

We found that CCCP treatment disturbed MMP, which was indicated by decreased intracellular Rho123 concentration (Figure 6). CCCP treatment decreased berberine accumulation in the HepG2 cells by up to 70% (*P*<0.01). The cellular concentration of berberine showed significant correlation (*P*<0.01, Pearson’s correlation coefficient is 0.914) with MMP that indicated by intracellular Rho123 concentration. The results indicated that MMP had huge effect on berberine accumulation in the HepG2 cells.

### Enhancive effects of MMP on berberine uptake

**Figure 7:**
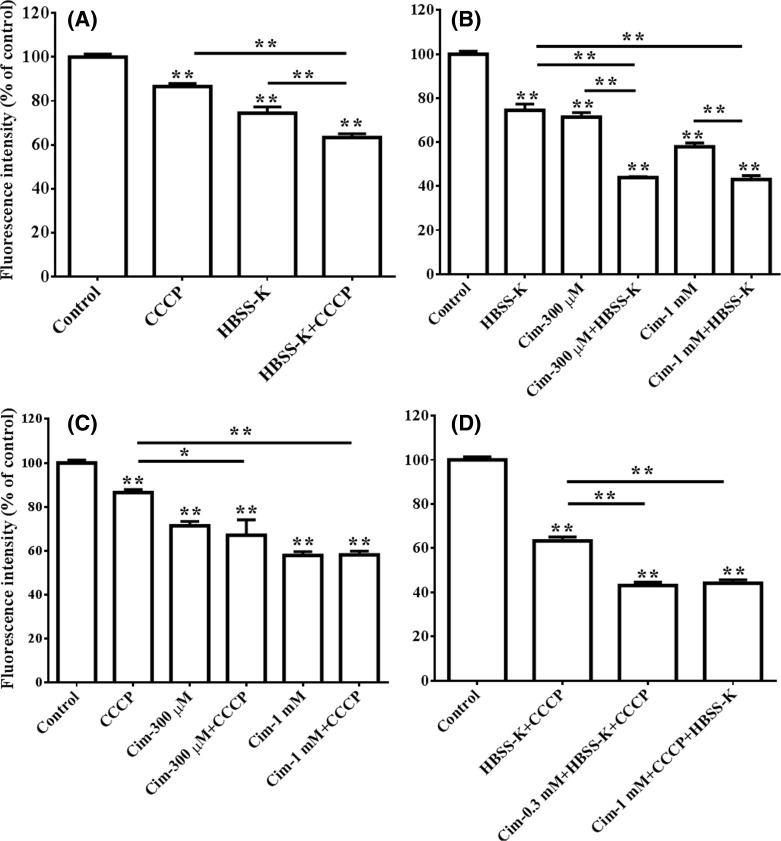
Enhancive effects of MMP on berberine uptake (Mean ± S.D., *n*=3) HepG2 cells were incubated with 10 μM berberine and with or without 0.125 μM CCCP, 0.3 or 1 mM cimetidine (Cim), or the HBSS-K solution 37°C for 0.5 h, respectively. After incubation, the cells were washed three times with chilled PBS and harvested by trypsinisation. The cells were stored in 0.5 ml chilled PBS and the concentration of berberine in the cells was quantified by flow cytometry analysis with excitation at 488 nm and emission at 530 nm. (**A**) Synergistic inhibition of CCCP and HBSS-K on berberine uptake in the HepG2 cells; (**B**) synergistic inhibition of HBSS-K and cimetidine on berberine uptake in the HepG2 cells; (**C**) influence of CCCP on the inhibition of HBSS-K in terms of berberine uptake in the HepG2 cells; (**D**) inhibition of the combination of CCCP, HBSS-K, and cimetidine on berberine uptake in the HepG2 cells.* indicates *P*<0*.*05 while ** indicates *P*<0.01 *versus* control or between groups indicated by the lines.

In terms of berberine uptake by the HepG2 cells, MMP enhanced the effects of cellular membrane potential (Figure 7A). In addition, cellular membrane potential enhanced the effects of cellular membrane expressed OCTs (Figure 7B). However, MMP did not enhance the effects of the OCTs (Figure 7C). The combination of CCCP, HBSS-K, and cimetidine (Figure 7D) showed the strongest inhibitory effects on intracellular berberine concentration in the HepG2 cells.

### Inhibitory effect of MMP on berberine efflux

**Figure 8:**
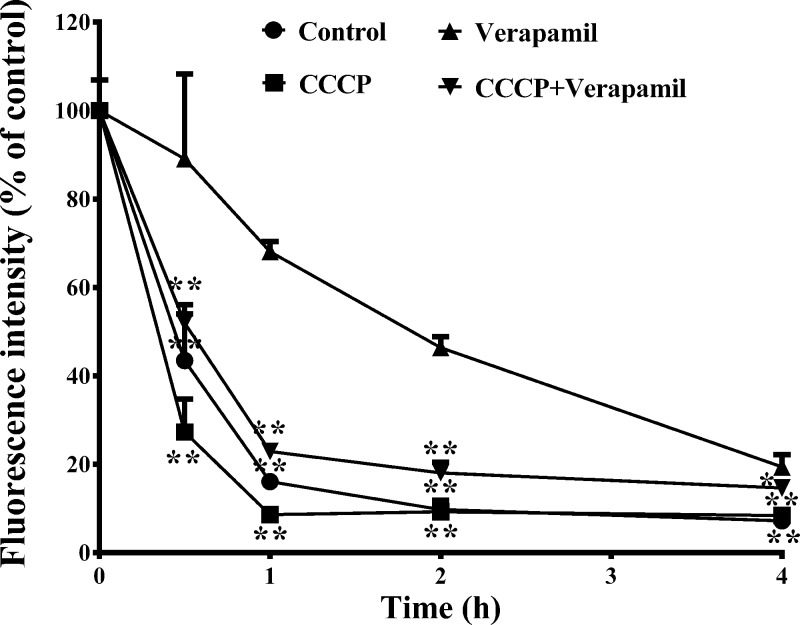
Inhibitory effect of MMP on berberine efflux (Mean ± S.D., *n*=3) HepG2 cells were pre-incubated with 10 μM berberine at 37°C for 2 h, then the cells were incubated with drug-free HBSS or HBSS containing CCCP (0.25 μM), or verapamil (100 μM), or CCCP (0.25 μM) plus verapamil (100 μM) at 37°C for 0.5, 1, 2, 4 h, respectively. After incubation, the cells were washed three times with chilled PBS and harvested by trypsinisation. The cells were stored in 0.5 ml chilled PBS and the accumulated berberine in the cells was quantified by flow cytometry analysis with excitation at 488 nm and emission at 530 nm. The amount of berberine was normalised to that at zero time point. * indicates *P*<0.05 while ** indicates *P*<0.01 *versus* verapamil treated groups.

As shown in Figure 8, the results of a two-way ANOVA indicated that berberine efflux was inhibited by verapamil (*P*<0.01); however, 0.25 μM CCCP significantly antagonised the inhibitory effect of verapamil (*P*<0.01) and restored the quick efflux of berberine. One-way ANOVA revealed the significant antagonistic effects of 0.25 μM CCCP on the inhibitory effect of verapamil at each time point.

## Discussion

The uptake by, subcellular distribution in, and efflux of berberine from the HepG2 cells were investigated in the present study. The results showed that berberine was accumulated in the HepG2 cells.

In normal liver cells, the uptake of berberine is exerted via drug transporters, mainly OCTs and OATPs [9,10]. However, owing to its low expression level in the HepG2 cells [16] and facilitative nature, the role of OCTs in berberine accumulation in the HepG2 cells is significant but limited, which was verified by the partial inhibitory effects of cimetidine at concentration of up to 1 mM in the present study. In addition, given that cimetidine inhibits mitochondrial respiration [27], which indicates potential inhibitory effects of cimetidine on MMP, the inhibitory effects of cimetidine on berberine accumulation was not totally generated from inhibiting the membrane OCTs. No significant inhibitory effect of rifampicin (a typical organic anion polypeptide transporter inhibitor [10]) was observed, which excluded the contribution of OATPs in the uptake of berberine by the HepG2 cells. Cell membrane potential had certain but weak effects on berberine uptake, as indicated by the slight inhibitory effect of HBSS-K, which is able to decrease the transmembrane potential difference. The results suggested that other crucial mechanisms were involved in the uptake and accumulation of berberine in the HepG2 cells.

The subcellular distribution of berberine was then directly observed using a LSCM benefiting from the auto fluorescence of berberine, and quantitatively analysed using an LC-MS/MS method. The results of experiments using CCCP showed that berberine was selectively accumulated in mitochondria owing to MMP. In addition, MMP was also crucial in berberine accumulation in human hepatocarcinoma HepaRG cells (data not shown, where CCCP also largely decreased the cellular concentration of berberine). Our results verified for the first time that mitochondrial distribution of berberine played central roles in the cellular accumulation of berberine in the hepatocarcinoma cells, which not only promoted the cellular entry but also inhibited the cellular efflux of berberine via an MMP-driven mechanism.

We assumed that the prompt and exhaustive uptake of berberine by mitochondria was beneficial to maintain the concentration gradient of berberine across the cell membrane, and the negative charge of the inner cell membrane; this, in turn, was helpful in enhancing the uptake of berberine mediated by cell membrane-expressed drug transporters, as well as, cell membrane potential. Furthermore, removing berberine from near the inner cell membrane was also helpful to reduce the cell membrane-expressed P-gp-mediated berberine efflux. However, the detailed mechanisms remain to be elucidated.

MMP is generated from a proton gradient across the mitochondrial inner membrane because of proton pumping by the respiratory chains located in this membrane [28]. Hence, the generation and maintenance of MMP is energy dependent, which explains the temperature-dependent uptake of berberine in HepG2 cells in the present study.

The accumulation of berberine in mitochondria might explain the wide and abundant tissue distribution of berberine [29]. For example, the exposure levels of berberine in the livers of rats [5] or mice [30] are respectively dozens or hundreds of times higher than that in circulation blood. However, the detailed mechanisms should be verified in the normal hepatocytes.

Mitochondria are dynamic organelles that are involved in a number of essential cellular processes. They play a focal role in energy metabolism as the major site where the cellular ATP is generated and are extensively involved in sickness and in health [31]. Mitochondrial accumulation of berberine shows specific inhibitory effect on respiratory complex I and thereby activate AMP-activated protein kinase (AMPK) [32]—a cellular energy sensor that exists in almost all eukaryotes. Once activated, AMPK switches off ATP-consuming, anabolic pathways, such as those involved in the synthesis of lipids, glucose, glycogen, and proteins [33]. Therefore, the activation of AMP kinases contributes to various pharmacological effects of berberine, including improvement of insulin action [32], lowering of blood lipid [34], reduction in atherosclerosis [35], stimulation of blood glucose uptake [36], combating aging and aging-related diseases [37], suppression of neuro-inflammatory responses [38], and inhibition of the metastatic potential of melanoma cells [39]. In terms of anti-tumour effects, mitochondria initiate apoptosis via a sequential process: mitochondrial membrane depolarisation, release of cytochrome *c*, activation of caspases, and final DNA fragmentation [40]. The mitochondrial accumulation of berberine explains its caspase-3/mitochondria pathway-dependent anti-tumour effects [14].

It should be noted that berberine reached its maximum concentration in mitochondria after 2 h of incubation but continued increasing for at least 6 h in whole cells in the present study. The results indicated the redistribution of berberine from mitochondria to other parts of the cells. It has been reported that berberine at low doses (12.5–50 μM) is concentrated in mitochondria but higher doses (over 50 μM) result in cytoplasmic and nuclear berberine accumulation [19]. It should be interesting to explore the underlying mechanisms that drive berberine transfer from one organelle to another.

Lysosome sequestering drugs were first described by De Duve et al. [41] more than 40 years ago. Drugs that are lysosomotropic share certain physicochemical properties, usually possessing a ClogP > 2 (ClogP, the calculated partition coefficient of the neutral species of the compound between octanol and water) and a basic p*K*_a_ (the logarithm of the dissociation constant of the most basic centre of the compound) between 6.5 and 11 [21]. Although berberine is a weak base, it was not sequestered by lysosomes in this study; this might be owing to its p*K*_a_, which is approximately 15.4 [1].

In conclusion, the results indicated that MMP played crucial roles in the accumulation of berberine in the HepG2 cells. This result explored the cellular pharmacokinetics of berberine in hepatocellular carcinoma cells and was helpful to reveal the potential site and mechanism of action.

## Abbreviations

AMPK: AMP-activated protein kinase
ANOVA: analysis of variance
CCCP: carbonyl cyanide 3-chlorophenylhydrazone
DMSO: dimethyl sulphoxide
ESI: electrospray ionisation
FBS: foetal bovine serum
HBSS-K: sodium-free HBSS
LC-MS/MS: liquid chromatography tandem mass spectrometry
LSCM: laser scanning confocal microscope
MMP: mitochondrial membrane potential
OATP: organic anion-transporting polypeptide
OCT: organic cation transporter
P-gp: p-glycoprotein
Rho123: rhodamine 123
